# A single-center prospective study on pain alleviation during peroral upper endoscopy with an ultrathin endoscope

**DOI:** 10.1186/s12876-023-02965-3

**Published:** 2023-09-21

**Authors:** Iwao Aya, Ryoji Ichijima, Tomomi Sugita, Masako Nakayama, Ayaka Takasu, Kanako Ogura, Takuji Gotoda, Hirofumi Kogure

**Affiliations:** 1 Division of Gastroenterology and Hepatology, Department of Medicine, Nihon University School of Medicine, 30-1, Oyaguchi Kami-cho, Itabashi-ku, Tokyo, 173-8610 Japan; 2 Health Management Center, Toranomon Hospital, Tokyo, Japan

**Keywords:** Transnasal ultrathin esophagogastroduodenoscopy), Upper GI tract, Reduction of discomfort undergoing upper endoscopy

## Abstract

**Background:**

The efficacy of transnasal endoscopy using an ultrathin endoscope has been reported in several studies. However, few studies regarding peroral endoscopy with ultrathin endoscopes with high resolution have been reported. This study investigates the pain alleviation of peroral endoscopy with an ultrathin endoscope.

**Methods:**

Patients with a history of peroral endoscopy using a conventional, normal-diameter scope with no sedation who underwent peroral esophagogastroduodenoscopy (EGD) using a thin scope between April-July 2022 were included in this study. After the procedure, the patients completed a questionnaire evaluating pain during the examination and willingness to repeat the procedure. The physicians were surveyed regarding their level of satisfaction. The primary endpoint was patient satisfaction, which corresponded to the rate of patients who rated the thin endoscope as more comfortable or somewhat more comfortable than the previously-used, conventional endoscope.

**Results:**

One hundred and forty-five patients were included in the analyses. Patient satisfaction was achieved in 86.2% (125/145) of patients. The median visual analog scale pain score was 3 (0–7) points in this study, which is significantly lower than the pain score after the previous endoscopy (5 (0–10) points; p < 0.001). In addition, 96% (24/25) of patients who underwent EGD by an expert and 95.8% (115/120) who underwent EGD by a non-expert were willing to repeat endoscopy using the thin scope (p = 0.69).

**Conclusion:**

Peroral endoscopy using a thin scope reduces patient pain regardless of the endoscopist’s experience.

## Background

Esophagogastroduodenoscopy (EGD) is important for the early detection of gastrointestinal tumors; however, the examination is often painful without sedation [1]. As pain lowers patients’ motivation to undergo repeat EGD, pain relief is important [2]. Endoscopy with sedation is expected to alleviate pain, though is associated with various adverse events, including respiratory depression and hypotension, which increase the need for careful monitoring, especially in older patients or patients with comorbidities [1]. Transnasal endoscopy results in less pain without the use of sedatives as it induces the gag reflex to a lesser extent than peroral endoscopy with a normal-diameter endoscope [3–11]. A suppressed gag reflex is useful during the Coronavirus disease-19 pandemic as it limits the spread of infection via air-borne droplets [12]. In addition, transnasal endoscopy does not significantly affect the patient’s hemodynamics, minimizing the burden on the patient [13, 14]. However, transnasal endoscopy may lead to nasal pain and bleeding as the scope is passed through the nasal cavity. The transnasal scope cannot be inserted in patients with a narrow nasal cavity. Thin endoscopes have poorer operability and imaging quality than conventional, normal-diameter scopes [15]. However, recent improvements to endoscopes have resulted in improved resolution [11]. The effectiveness of peroral endoscopy using a thin scope remains unclear (5,16,18), and no studies regarding the effects of pain relief during peroral endoscopy using an ultrathin scope in patients who previously underwent endoscopy using a normal-diameter scope have been reported. Therefore, this study evaluates the safety and pain-relieving efficacy of peroral endoscopy using an ultrathin scope in patients with a history of peroral endoscopy using a normal-diameter scope.

## Methods

### Study design

This single-center, single-arm, prospective study was registered with the University Hospital Medical Information Network (000047366) and conducted in accordance with the Declaration of Helsinki with the written consent of all patients. Approval was obtained from the institutional review board of Nihon University Hospital.

**Fig. 1:**
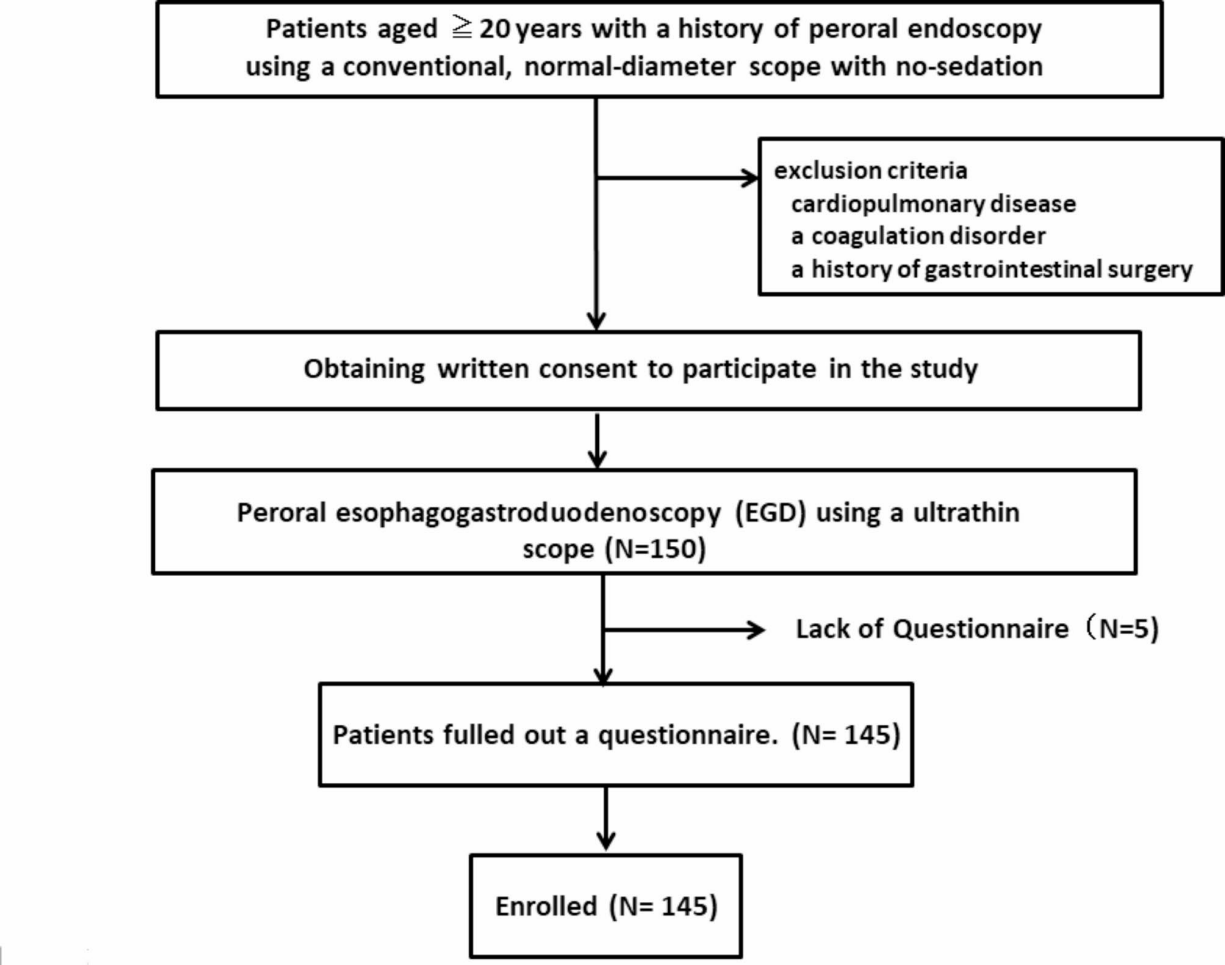
Flowchart of this study

Figure 1 shows a summary of this study flow chart on patients enrollment. Patients aged ≥ 20 years with a history of peroral EGD using a normal-diameter scope without intravenous anesthesia who were scheduled for upper endoscopy between April-July 2022 were included in the study. All included patients provided informed written consent for their participation in this study. Patients with cardiopulmonary disease, a coagulation disorder, or a history of gastrointestinal surgery were excluded from the study.

Patients were instructed to begin fasting at 21:00 the day before EGD. Lidocaine spray (Xylocaine Pump Spray 8%; Sandoz KK, Tokyo, Japan) was applied to the throat for local anesthesia as a pre-endoscopy treatment. An ultrathin endoscope with a 5.4 mm diameter (GIF-XP290N, Olympus Medical System, Tokyo, Japan) or 5.8 mm diameter (EG-740 N, FUJIFILM Corporation, Saitama, Japan) was used. All EGD examinations were performed by one of eight endoscopists with five or more years of endoscopy experience as a gastroenterologist. Three endoscopists were considered experts, while five were non-experts. Operators who were certified by the Japan Gastroenterological Endoscopy Society as endoscopy specialists were defined as experts.

After the procedure, the patients evaluated the pain experienced during the previous endoscopy (using a normal-diameter scope) and that during the present endoscopy (using the ultrathin scope) on a five-point scale (1, more comfortable than the previous endoscopy; 2, somewhat more comfortable than the previous endoscopy; 3, same as the previous endoscopy; 4, more uncomfortable than the previous endoscopy; and 5, significantly more uncomfortable than the previous endoscopy). The patients also rated their throat discomfort during the previous and present endoscopies using the visual analog scale (0, no pain through 10, worst pain imaginable). The patients also reported their willingness to undergo repeat endoscopy using the same scope and their preference for a thin scope during endoscopy. The physicians also completed a questionnaire after the procedure regarding their satisfaction with the endoscopy. The satisfaction defined as: A, ease of endoscopy without complaints; B, uneasiness with endoscopy due to reflex or operability issues; or C, unsatisfactory endoscopy requiring a different scope or future endoscopy with sedation. A (ease of endoscopy without complaints) was defined as physician satisfaction.

### Sample size calculation

In a previous study, when upper EGD was performed using an ultrathin endoscope and a normal-diameter endoscope, 31% of patients who underwent EGD with the normal-diameter scope and 14% of patients who underwent EGD with the ultrathin scope requested sedation during the next endoscopy [16]. Based on this report, a threshold efficacy probability of 0.15, an expected efficacy probability of 0.25, an α of 0.05, and 1-β of 0.9, this study required 136 patients. Based on a drop-out rate/exclusion rate of 10%, the target number of patients was set at 150.

### Study endpoint

The primary endpoint was patient satisfaction, which was defined as the rate of patients who answered that the present endoscopy was more comfortable or somewhat more comfortable than their previous peroral endoscopy. A subgroup analysis was conducted to compare the differences in the primary endpoint based on the endoscope and level of endoscopist expertise.

The secondary endpoints included the physicians’ satisfaction with the thin endoscope, patients’ rate of willingness undergo repeat endoscopy using the thin endoscope, patient pain score, EGD time, biopsy success rate and time, and tumor detection rate.

We also performed a sub-analysis of patients with a pain score of 5 or higher in the previous examination. We evaluated the rate of improvement in pain compared to the previous endoscopy and the rate of willingness undergo repeat endoscopy using the ultrathin scope.

### Statistical analysis

Continuous variables are presented as median and range. Categorical variables are presented as number and frequency. Wilcoxon’s rank sum test was used to compare the continuous variables, and Fisher’s test was used to compare the categorical variables. Statistical significance was set at p < 0.05. All statistical analyses were conducted using JMP (version 13.0.0, SAS Institute, Cary, NC, United States).

## Results

Of the 150 patients enrolled in this study between April 1 and July 31, 2022, five were excluded due to incomplete questionnaire entries (Fig. 1). The final analyses included 145 patients.

The median patient age was 60 years (36–87 years), and 104 patients were men (Table 1). Expert endoscopists performed EGD in 25 patients (17.2%), and non-expert endoscopists performed EGD in 120 patients (82.8%). The EG-740 N scope was used in 88 patients (60.7%), and the GIF-XP290N scope was used in 57 patients (39.3%). No adverse events were reported. The median EGD time was 5 min (range: 2–8 min). Thirty-nine patients (6.9%) underwent biopsy at the time of endoscopy, and the median biopsy time was 14 s (range: 5–38 s). The biopsy success rate was 100%, and the tumor detection rate was 0%.

**Table 1:** Patient characteristics (n = 145)

Age (years)	60 (36–87)
Sex, (male / female)	104 (71.7%) / 41 (28.3%)
Procedure indications Cancer screening Follow-up examination for atrophic gastritis Screening for varices Screening for anemia Gastroesophageal reflux	68 (46.9%) 25 (17.2%) 17 (11.7%) 10 (6.9%) 25 (17.2%)
Expert / Non-expert endoscopist	25 (17.2%) / 120 (82.8%)
EG-740 N / GIF-XP290N	88 (60.7%) / 57 (39.3%)
Complete examination	145 (100%)
EGD time (min)	5 (2–8)
Adverse events	0 (0.0%)
Biopsy	39 (6.9%)
Successful biopsy	39 (100%)
Biopsy time (s)	14 (5–38)

**Table 2:** Procedure characteristics

	Peroral endoscopy with an UT-EGD n = 145	Previous non-sedated C-EGD n = 145	*P-value
EGD time (min)	5 (2–8)	4 (1–14)	< 0.01
Patient satisfaction	125 (86.2%)		
Pain score	3 (0–7)	5 (0–10)	< 0.01
Physician satisfaction	111 (76.6%)		
Willing to repeat peroral endoscopy with an UT-EGD	139 (95.9%)		0.74

Data are presented as median (range) or number (percentage)

Abbreviations: UT-EGD, ultrathin esophagogastroduodenoscopy; C-EGD, conventional esophagogastroduodenoscopy;

*P-value calculated using the chi-square test or Fisher’s exact test for categorical data

or a t-test or the Mann–Whitney U test for continuous data

When expert endoscopists performed the procedure, the median pain score was 2 points (range: 0–6 points), which was significantly lower than the median pain score when the procedure was performed by non-expert endoscopists (median: 3 points; range: 0–7 points; p = 0.04) (Table 3). The patient satisfaction rate was higher when expert endoscopists performed the procedure (88%, 22/25) than when non-expert endoscopists performed the procedure (85.8%, 103/120), though the difference was not significant (p = 0.53). Most patients who underwent endoscopy by an expert (96%; 24/25) or a non-expert (95.8%; 115/120) were willing to undergo repeat endoscopy using the same scope (p = 0.69)

**Table 3:** Patient satisfaction when the procedure was performed by an expert or non-expert endoscopist

	Expert endoscopist n = 25	Non-expert endoscopist n = 120	*P-value
Patient satisfaction	22 (88.0%)	103 (85.8%)	0.53
Pain score	2 (0–6)	3 (0–7)	0.04
Physician satisfaction	19 (76.0%)	92 (80.0%)	1.00
Willing to repeat peroral endoscopy with an UT-EGD	24 (96.0%)	115 (95.8%)	0.69

Data are presented as median (range) or number (percentage)

Abbreviations: UT-EGD, ultrathin esophagogastroduodenoscopy

Expert endoscopists are defined as those who have been certified by the Japan Gastroenterological Endoscopy Society as endoscopy specialists

*P-value was calculated using the chi-square test or Fisher’s exact test for categorical data and the

t-test or the Mann–Whitney U test for continuous data

The pain score, patient satisfaction rate, or patient willingness to undergo repeat endoscopy were not significantly different between patients who underwent EGD using the EG-740 N or GIF-XP290N endoscope (Table 4)

**Table 4:** Patient satisfaction when the procedure was performed using EG-740 N or GIF-XP290N endoscope

	EG-740 N n = 88	GIF-XP290N n = 57	*P-value
Patient satisfaction	76 (86.3%)	49 (86.0%)	1.00
Pain score	3 (0–6)	3 (0–7)	0.82
Physician satisfaction	65 (73.9%)	46 (80.7%)	0.42
Willing to repeat peroral endoscopy with an UT-EGD	84 (95.5%)	55 (96.5%)	1.00

Data are presented as median (range) or number (percentage)

Abbreviations: UT-EGD, ultrathin esophagogastroduodenoscopy

EG-740 N: An ultrathin endoscope with a 5.8 mm diameter

GIF-XP290N: An ultrathin endoscope with a 5.4 mm diameter

*P-value was calculated using the chi-square test or Fisher’s exact test for categorical data or the

t-test or the Mann–Whitney U test for continuous data

## Discussion

This study evaluated the patient and physician satisfaction with peroral endoscopy using an ultrathin scope in Japanese patients. While several studies regarding pain relief with the use of transnasal endoscopy have been reported, few studies have examined pain relief via the use of a thin endoscope during peroral endoscopy [5, 16, 17]. Furthermore, all studies regarding the use of an ultrathin endoscope during peroral endoscopy include outdated endoscopes. In this study, new, thin endoscopes with improved resolution were used. The patient and physician satisfaction rates were compared when an expert or a non-expert endoscopist performed the procedure. Based on the patient satisfaction rate and pain scores reported in this study, the new, thin endoscopes resulted in reduced pain for the patients. Furthermore, patients in whom the procedure was conducted by an expert endoscopist reported lower pain scores than those in whom the procedure was conducted by a non-expert endoscopist, though the patient satisfaction and willingness to undergo a repeat procedure were not different between the groups. These results suggest that peroral endoscopy using a thin endoscope alleviates patient pain regardless of the endoscopist expertise level. Furthermore, no adverse events were reported in this study, the physician satisfaction rate was high, and all attempted biopsies were performed easily. Sub-analysis results showed that most patients (90% or more) who had previously experienced pain were able to relieve their pain, and the rate of requesting re-examination was high. This is clinically important and suggests the utility of peroral endoscopy using an ultrathin endoscope.　 On the other hand, 20 patients indicated that this procedure was unsatisfactory. Twelve of the twenty patients had an EGD time longer than the previous EGD time. In addition, only one patient had less pain than that during the previous EGD. Since patients were informed in advance that a thinner scope than the previous one would be used, they may have felt dissatisfied that the pain had not reduced.

A previous study reported no difference in pre-endoscopy anxiety, intra-endoscopy pain, gag reflex, or general tolerability between patients who underwent peroral and transnasal endoscopy using the same thin scope without sedation (18). However, the pain prior to the scope passing through the throat was higher among patients who underwent transnasal endoscopy. Another study reported more favorable outcomes after transnasal endoscopy, including better general tolerability, tolerability as the scope passed through the throat, and willingness to undergo repeat endoscopy with the same endoscope [5]. However, the previous studies did not compare peroral and transnasal endoscopy, though the fact that the pain was better controlled during transnasal endoscopy when a thin endoscope was used suggests that the use of a thin endoscope during peroral endoscopy will also reduce the pain. Furthermore, a previous study compared the use of a thin (6.0 mm) and a normal-diameter (9.8 mm) endoscope during unsedated EGD and reported significantly lower pain while passing through the throat and during the procedure and a significantly lower rate of patients who desired sedation for the next endoscopy among patients who underwent thin endoscopy [16]. The results of these previous studies are consistent with those of the current study, though the operability of the endoscope to perform a biopsy and the outcomes based on endoscopist expertise were only investigated in the current study.

The tumor detection rate in this study was 0%. This may be due to the facts that a history of peroral endoscopy was an eligibility criterium of this study, the patients were relatively young and underwent endoscopy as part of routine screening, or the study included a low rate of patients with atrophied mucosa (37.2%, 54/145).

Peroral endoscopy using a thin scope has several advantages, including its cost performance, safety, and low pain scores without the use of sedation. The use of a thin endoscope during peroral endoscopy reduces the pain score regardless of the endoscopist expertise as nasal pain is avoided. In addition, increasing the willingness of patients to undergo repeat endoscopy may lead to increased rates of early cancer detection, which is especially important in patients with no mucosal atrophy in whom gastric cancer may be detected. These results highlight the importance of a screening endoscopy method that minimizes the pain for the patient.

This study is not without limitations. First, it was a single-center, single-arm study with a small sample size. Although the patient satisfaction was compared to that after the previous peroral endoscopy with a normal-diameter scope, this was not a randomized controlled study. Therefore, the evaluation index of the study is subjective. Since the subjects signed an informed consent form before participating in the trial and had relevant understanding of the trial situation, this may have affected the trial results due to psychological implications. Second, this study only included patients with a history of peroral endoscopy using a conventional, normal-diameter scope with no sedation. This may have resulted in a selection bias, as patients who have had previous painful experiences with endoscopy or who require sedation during the procedure may not have been included in the study. Third, the evaluation indicators of this study were subjective and may have recall bias. Fourth, the endoscopy procedures may have been performed by different endoscopists. Finally, although the physician satisfaction level was high, the imaging quality of the thin scope was not evaluated in this study. Image resolution affects the malignancy detection rate.

Although this study suggested the advantage of peroral endoscopy with an ultrathin endoscopy, it is difficult to conclude that from this study alone, since patients who experienced severe distress with conventional endoscopy in the past may have been excluded from this study because they avoided undergoing endoscopy again.

## Conclusion

The use of a ultrathin endoscope during peroral endoscopy reduces patient pain.　A multi-center, prospective study comparing the cancer detection rate of EGD using a normal-diameter endoscope with that using a ultrathin endoscope is necessary

Table 5 shows the result of sub-analysis of patients with a pain score of 5 or higher in the previous examination. A total of 66.9% (97/145) of the patients reported that their pain score during their previous examination was 5 or higher. Of these, 75.3% (73/145) were men and 24.7% (24/145) were women. The difference in the median pain score between the previous and current examination was significant (7 points vs. 3 points, P < 0.01). Compared to the previous EGD, 91.8% (89/97) of patients had improved pain scores. Additionally, 97.0% (95/97) of patients were willing to undergo repeat peroral endoscopy with an UT-ESD.

**Table 5:** Characteristics of patients with a pain score of 5 or higher during their previous examination

	Peroral endoscopy with	Previous non-	*P-value
	an UT-EGD	sedated C-EGD	
	n = 97	n = 97	
EGD time (min)	4 (2–8)	4 (1–9)	0.101
Pain score	3 (0–7)	7 (5–10)	< 0.01
EG-740 N / GIF-XP290N	59 (60.8%) / 38 (39.2%)		
Willing to repeat peroral endoscopy with an UT-ESD	95 (97.0%)		

Data are presented as median (range) or number (percentage)

Abbreviations: UT-EGD, ultrathin esophagogastroduodenoscopy; C-EGD, conventional esophagogastroduodenoscopy;

*P-value calculated using the chi-square test or Fisher’s exact test for categorical data

or a t-test or the Mann–Whitney U test for continuous data

## Data Availability

The datasets used and/or analyzed during the current study are available from the corresponding author on reasonable request.

## Abbreviations

EGD: Esophagogastroduodenoscopy
UT-EGD: Ultrathin esophagogastroduodenoscopy
C-EGD: Conventional esophagogastroduodenoscopy
